# Pediatric acute myeloid leukemia with breast chloromas

**DOI:** 10.1002/jha2.669

**Published:** 2023-03-15

**Authors:** Maria S. Gallardo, Richard Joyrich, Jeffrey W. Taub

**Affiliations:** ^1^ Division of Pediatric Hematology/Oncology Children's Hospital of Michigan Detroit Michigan USA; ^2^ Department of Hematology‐Oncology Karmanos Cancer Institute, Wayne State University Detroit Michigan USA; ^3^ Discipline of Pediatrics Central Michigan University College of Medicine Detroit Michigan USA; ^4^ Department of Pediatrics Wayne State University School of Medicine Detroit Michigan USA

1

Myeloid sarcoma (MS; also known as granulocytic sarcoma or chloroma) is a solid extramedullary mass composed of primitive myeloid cells that disrupts the normal architecture of the tissue in which it is found. MS can present concurrently with acute myeloid leukemia (AML), precede the diagnosis of AML without evidence of leukemia in the blood or bone marrow or as a relapse in a patient with previously treated AML [1, 2, 3]. In children, the overall incidence of MS in association with AML varies, but has been reported to be as high as 24% [4, 5, 6, 7, 8]. The most frequently involved anatomic sites are the skin, lymph node, testis, gastrointestinal tract, bone, and central nervous system (CNS) [1, 2, 3, 9, 10].

Breast MS is very rare and only accounts for approximately 8% of MS cases [1, 10], with the majority of cases of breast MS diagnosed in adults [11, 12, 13, 14, 15, 16, 17, 18, 19, 20, 21]. There have been three reported cases in adolescents with previously treated AML [22, 23, 24] and only one case of concurrent diagnosis of a breast MS at the time of AML diagnosis [25]. We report a case of bilateral breast MS in a teenage Asian American patient diagnosed concurrently with AML.

An 11‐year‐old teenage female patient (Asian‐Indian descent) presented with a 1–2‐month history of generalized pallor and fatigue. She was also noted to have a 1‐month history of bilateral breast masses. On presentation, her complete blood count demonstrated a white blood cell count of 24,800/mm^3^, hemoglobin level of 3.9 g/dL, and platelet count of 19,000/mm^3^. A bone marrow aspirate/biopsy demonstrated near replacement with sheets of cells with high N/C ratio and round nuclei with single nucleolus present. Auer rods were not present. Erythroid and megakaryocytic elements were decreased, and myeloid elements showed decreased maturation. CD34 and CD117 highlighted approximately 50% of cells. Myeloperoxidase was strongly positive in the majority of cells.

Flow cytometry revealed that the blasts expressed CD34, CD13, CD33, CD117, and CD40. She was classified as CNS [2]. Chromosomal analysis demonstrated 46,XX,der(21)t(8;21)(q22;q22)ins(5;21)(q12q21;q22) [20], and fluorescence in situ hybridization (FISH) analysis demonstrated RUNX1–RUNX1T1/t(8;21) variant gene fusion in 98.5% of cells and gain of chromosome 15q24 in 7% of cells.

She underwent bilateral breast biopsies which showed pleomorphic cells with scant pale cytoplasm and irregular nuclear contours. The pleomorphic cells were CD34 positive and had focal CD117 immunoreactivity. FISH analysis demonstrated the RUNX1/RUNX1T1 t(8;21) variant gene fusion.

A positron emission tomography–computed tomography (PET/CT) scan demonstrated several nodular masses in both breasts with mild fluorodeoxyglucose (FDG) uptake, thickening over the left chest wall with focal increased FDG uptake, presacral soft tissue mass with hypermetabolic activity, and intense FDG uptake in the upper sacrum and sub‐centimeter subcarinal lymph node with mild FDG uptake.

She was treated on the St. Jude Children's Research Hospital AML16 clinical trial “A Phase II Trial of Epigenetic Priming in Patients with Newly Diagnosed Acute Myeloid Leukemia” [NCT03164057]. She received a total of two induction and two intensification cycles of therapy using the drugs decitabine, cytarabine, daunorubicin, etoposide, fludarabine, and mitoxantrone.

A repeat PET scan performed after the induction I cycle demonstrated improved appearance of the breast masses with decreasing FDG tracer uptake overall, focal area of soft tissue thickening in the left chest wall was no longer evident and improved subcarinal adenopathy (Figure 1). A PET scan performed at the end of therapy (i.e., after intensification II) demonstrated continued appearance of mild increased FDG tracer uptake in the lateral aspect of the right breast with no evidence for FDG avid adenopathy.

**FIGURE 1:**
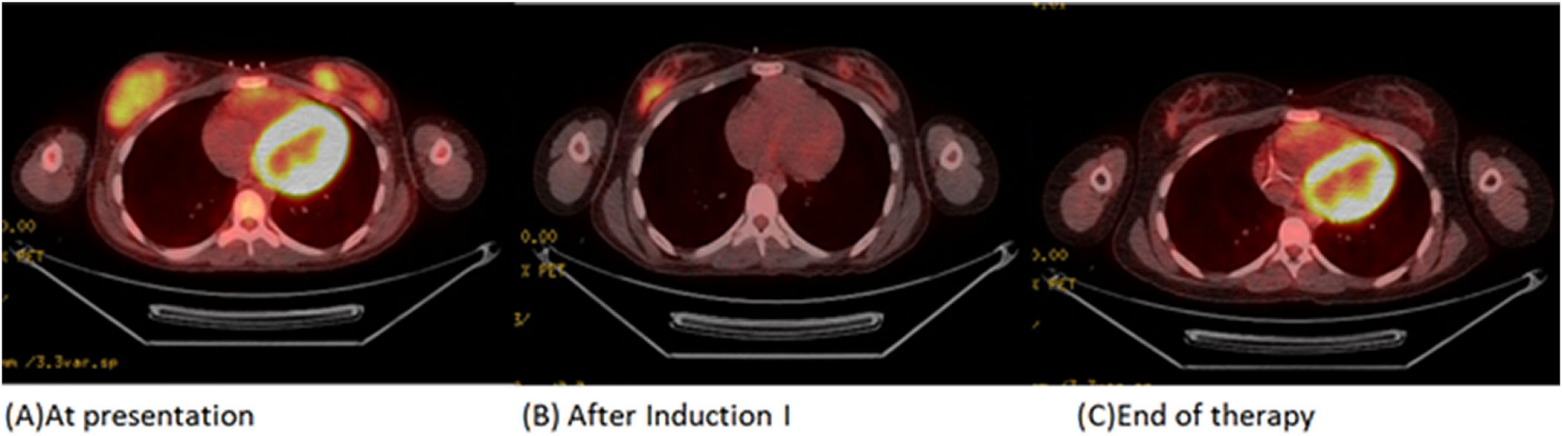
(A) Several nodular masses demonstrated in both breasts with fluorodeoxyglucose (FDG) uptake (standardized uptake value [SUV] max: 2.9 on right breast and SUV max: 2.7 on left breast), thickening over the left chest wall between the posterior medial fifth and sixth ribs with focal increased FDG uptake, and intense FDG uptake in the upper sacrum and sub‐centimeter subcarinal lymph node with mild FDG uptake. (B) Improved appearance of the breast masses with decreased FDG uptake overall (SUV max: 2.1 on right breast and SUV max: 1.9 on left breast), focal area of soft tissue thickening in the left chest wall between the medial fifth and sixth ribs was no longer evident and no longer abnormal tracer uptake in that region, and improved subcarinal adenopathy. (C) No area of abnormal tracer uptake is seen on the left breast but continued appearance of mild increased FDG tracer uptake in the lateral aspect of the right breast (SUV max: 1.8) with no evidence for FDG avid adenopathy.

Her last bone marrow minimal residual disease (MRD) analysis performed 6 months following the completion of therapy was negative (<0.1%) and FISH analysis to detect the RUNX1–RUNX1T1/t(8;21) gene fusion was negative. She currently remains in complete remission >1.5 years off therapy.

MS clinical features of breast granulocytic sarcoma (GS) are nonspecific, which can present as a unilateral or bilateral palpable, painful, or painless breast mass/nodules and often can be indistinguishable from benign tumors or lymphoma [2, 10], making the diagnosis challenging. A tissue biopsy with immunohistochemical stains for the antigens most frequently expressed by MS, including CD34, CD43, CD56, MPO, CD117, CD13, and CD33 [2, 3] is important. PET/CT imaging appears to be the best imaging modality to assess the presence of extramedullary AML, which has ability to both detect MS and monitor its response to therapy [26, 27].

There is no consensus about the optimal treatment of MS. High‐dose chemotherapy, radiation, surgical resection, and allogeneic stem cell transplantation are all modalities that can be incorporated into the therapy of MS [1, 27]. It appears appropriate to treat chloroma with AML‐type chemotherapy protocols even in the absence of systemic manifestations since AML is almost always present [28].

Multiple chromosomal anomalies are associated with extramedullary leukemia, and these are reported in about 54%–70% of patients [26]. Common abnormalities include t(8,21) (q22;q221) RUNX1–RUNXT1, NPM1 mutation, inv(16), 11q23, t(9;11), t(8;17), t(8;16), t(8;17), t(1;11), trisomies of chromosomes 4, 8, 11, monosomy 7, and deletions of chromosomes 5q, 16q, and 20q [9, 10, 28].

The prognostic significance of MS in childhood AML is still controversial. Several groups have reported that complete remission rates were lower in patients with extramedullary infiltration following induction chemotherapy [7, 8, 29], while others reported that overall survival and event‐free survival rates were better among patients with AML and MS [4, 5, 30].

The characteristics of our patient are similar to those of the Tyagi study [6] who reported an increased frequency of t(8;21), 29.9% of 472 Indian children studied were positive for t(8;21). Additionally, a significant association of t(8;21) with chloromas (*p* < 0.01) was observed in northern Indian children with AML. This compares well with one study which reported a similar frequency (26%) of positive t(8;21) among their 567 pediatric patients from a tertiary care cancer center from western India [31].

Chloromas involving the breast in an adolescent female patient as initial presentation for AML is highly unusual and remains a diagnostic challenge; therefore, it must be included in the differential diagnosis of a breast mass in children and adolescents with or without a previous history of leukemia.

## AUTHOR CONTRIBUTIONS

Maria S. Gallardo drafted and edited the manuscript. Richard Joyrich edited the manuscript. Jeffrey W. Taub planned the report and edited the manuscript.

## CONFLICT OF INTEREST STATEMENT

The authors have no conflicts of interest.

## FUNDING INFORMATION

The authors received no specific funding for this work.

## PATIENT CONSENT STATEMENT

Verbal consent was obtained from patient and family.

## Data Availability

Data sharing is not applicable to this article as no new data were created or analyzed in this study.
